# Hypoglycemic and hypolipidemic effects of *Bersama engleriana* leaves in nicotinamide/streptozotocin-induced type 2 diabetic rats

**DOI:** 10.1186/1472-6882-12-264

**Published:** 2012-12-26

**Authors:** Watcho Pierre, Achountsa Jeugo Hugues Gildas, Mbiakop Carlos Ulrich, Wankeu-Nya Modeste, Nguelefack Télesphore Benoît, Kamanyi Albert

**Affiliations:** 1 Animal Physiology and Phytopharmacology Laboratory, Department of Animal Biology,Faculty of Science, University of Dschang, P.O. Box 67, Dschang, Cameroon

**Keywords:** Bersama engleriana, Diabetes mellitus, Hypoglycemic, Hypolipidemic, Rat

## Abstract

**Background:**

The present investigation was aimed at evaluating the hypoglycemic and hypolipidemic properties of the aqueous and methanolic extracts from *Bersama engleriana* leaves in streptozotocin/nicotinamide (STZ-NA)-induced type 2 diabetic rats.

**Methods:**

Animals were orally treated for 4 consecutive weeks with *Bersama engleriana* extracts at doses of 300 or 600 mg/kg. The anti-diabetic effect was examined by measuring blood glucose (BG) at 0, 1, 14 and 28 days after STZ-NA treatment and, total cholesterol (TC), high density lipoprotein cholesterol (HDL-C), low density lipoprotein cholesterol (LDL-C) and triglycerides (TG) levels at sacrifice (day 29). Glibenclamide (0.25 mg/kg) was used for comparison.

**Results:**

STZ-NA-induced diabetic rats showed moderate to significant increases in the levels of BG, TG, TC, LDL-C while body weight, HDL-C levels and relative weights of liver and pancreas were decreased compared to controls (non diabetic rats). Administration of the plant extracts to STZ-NA diabetic rats resulted in a significant decrease in BG, TG, TC and LDL-C and the dose 600 mg/kg of the methanolic extract was the most effective; HDL-C level was markedly increased after four weeks compared to untreated diabetic rats. A dose-dependent increase in the relative weights of the diabetogenic organs was observed in the *Bersama engleriana* groups. It can be also noticed that the methanolic extract, especially the dose 600 mg/kg (p<0.001), produced more effects than glibenclamide and aqueous extract. Rats treated with glibenclamide (0.25 mg/kg) generally gave lower results compared to groups treated with plant extracts.

**Conclusion:**

Results of the present study showed that *Bersama engleriana* extracts and especially its methanolic extract possess antidiabetogenic properties and beneficial effects on diabetic hyperlipidemia. All these effects could be due to the bioactive components revealed in the *Bersama engleriana* extracts such as triterpenes and phenols and which could justify its ethnomedical use.

## Background

Type 2 diabetes is caused by the failure of beta cells to compensate for insulin resistance. This leads to hyperglycaemia, which can in turn exert deleterious effects on β cells [1]. Evidence for the importance of plant extracts in the management of type 2 diabetes is emerging. Medicinal plants are frequently considered to be less toxic and free from side effects than synthetic ones. *Bersama engleriana Gurke* (Melianthaceae) is one of such plants; it is a small or medium size tree of 6-9 m, rarely exceeding 25 m in height. It is widespread throughout tropical Africa, preferring higher rainfall or evergreen forests. It is distributed from Senegal to Zaire, and parts of southern Africa; the leaves are used for the treatment of many ailments including diabetes and male impotence [2].

Previous findings have exhibited a variety of pharmacological potentials including blood glucose lowering concentration in oral glucose tolerance test [3], aphrodisiac [4], non-toxic, anti-tumor, antioxidant and antimicrobial [5] and, ejaculation delaying (unpublished data) activities. The present work was undertaken to investigate the antidiabetic effects of aqueous and methanol extracts from the leaves of *Bersama engleriana* in a non-obese diabetes model characterized by reduced pancreatic insulin contents and moderate hyperglycemic levels, like those usually occurring in human type 2 diabetes. This model, obtained by the combined administration of nicotinamide (NA) and streptozotocin (STZ) is increasingly used for pharmacological research in diabetes [6-9].

## Methods

### Collection of plant material and extraction

The leaves of *Bersama engleriana* (Melianthaceae) were collected in the Bamboutos mountains, west region of Cameroon. Botanical identification was carried out at the National Herbarium, Yaoundé, Cameroon (HNC) where a voucher specimen No. 32427/HNC has been deposited. The leaves were shade-dried and reduced to powder.

### Aqueous extract

A total of 400 g of the powder of *Bersama engleriana* leaves were extracted in 5 l of distilled water for 1 h and boiled for 30 min. The heated decoction was taken and allowed to cool at room temperature (22 ± 2°C). The decoction was filtered and the filtrate was oven dried (45°C). The resulting material was found to weigh 112 g (28% yield, w/w based on the dried starting weight). The working solution was obtained by dissolving 1 g of the residue in a known volume of distilled water and the final volume adjusted to 10 mL.

### Methanolic extract

Ground leaves (600 g) of *Bersama engleriana* were mace-rated with methanol (3 L; 2 × ) for 72 h to yield, after solvent evaporation under reduced pressure, 16.5 g of brownish extract corresponding to an extraction yield of 14.29% (w/w based on the dried starting weight). The working methanolic extract was obtained by dissolving 1 g of the residue in a known volume of distilled water and the final volume adjusted to 10 mL.

### Preliminary phytochemical screening

Qualitative phytochemical evaluation was performed on aqueous and methanolic extracts of the leaves of *B. engleriana* to determine the presence or not of flavonoids (test of Shinoda), sterols (Libermann Buchard test), phenols (ferric chloride test), alkaloids (Dragendorff test), saponins (saponification test) all these tests were performed as described by [10].

### Animals

Adult male Wistar rats weighing 200-300 g were used in this study. The animals were maintained at room temperature (22-23°C) with a reverse natural light-dark cycle in the animal house of the Faculty of Science, University of Dschang, Cameroon. Food and tap water were available *ad libitum*. The experiments were performed in accordance with the internationally accepted standard ethical guidelines for laboratory animal use and care as described in the European Community guidelines; EEC Directive 86/609/EEC, of the 24th November 1986 [11].

### Induction of diabetes

Nicotinamide (95 mg/kg b.w.) (Sigma, Saint-Louis, MO, USA), dissolved in saline, was injected intraperitoneally 15 min before administration of STZ (60 mg/kg b. w.) (Sigma, Saint-Louis, MO, USA), which was dissolved in buffer citrate (pH 4.5) immediately before use. After 1 week, rats with moderate diabetes having hyperglycemia with blood glucose ≥ 126 mg/dl were used for the study [12]. Blood was collected from the tail vein. Controls received both vehicles.

### Experimental design

In the experiment, a total of 42 rats (36 diabetic rats + 6 normal rats) were used. Diabetes was induced in rats, 2 weeks before starting the treatment. The animals were divided into seven groups containing six rats each and distributed as follows:

Group 1, control rats given distilled water (10 ml/kg b.w.); Group 2, diabetic rats given distilled water (10 ml/kg b.w.); Group 3, diabetic rats given glibenclamide (0.25 mg/kg b.w.); Group 4, diabetic rats given aqueous extract of *Bersama engleriana* (300 mg/kg b.w.); Group 5, diabetic rats given aqueous extract of *Bersama engleriana* (600 mg/kg b.w.); Group 6, diabetic rats given methanolic extract of *Bersama engleriana* (300 mg/kg b.w.); Group 7, diabetic rats given methanolic extract of *Bersama engleriana* (600 mg/kg b.w.). All test substances were administered by gastric gavage for 4 weeks. Glycaemia was measured 0, 1, 2, and 4 weeks after pharmacological treatments. Body weight and relative weights of pancreas and liver were recorded. All the seven groups of rats were killed one day after the last treatment (day 29) by cervical dislocation. Blood was collected for total cholesterol (TC), HDL-cholesterol (HDL-C), LDL-cholesterol (LDL-C), and triglycerides (TG) estimations using commercial kits from INMESCO (Germany).

### Statistical analysis

Data are expressed in mean ± SEM due to the non homogeneity of groups. The effect of the duration of the treatment (week 0 to week 4) on blood glucose concentration was analyzed using ANOVA Repeated Measures followed by Bonferroni test when necessary. For other parameters, One-way analysis of variance (ANOVA) followed by post-hoc Bonferroni was performed using SPSS for Windows version 12.0. Comparisons with p values < 0.05 were considered to be statistically significant.

## Results

### Phytochemical analysis

Qualitative phytochemical screening of the aqueous and methanolic extracts of the leaves of *Bersama engleriana* showed sterols, triterpens and saponins (Table 1).

**Table 1:** Results of the phytochemical screening of *Bersama engleriana *extracts

**Group of compounds**	**Aqueous extract**	**Methanolic extract**
Flavonoids	-	-
Sterols	+	+
Phenolic compounds	-	-
Alkaloids	-	-
Saponins	+	+

**+**: present - : absent.

### Effects of Bersama engleriana on body weight and relative organ weights

Induction of diabetes (untreated diabetic rats) significantly (p<0.01) decreased the body weight compared to controls.

Oral treatment of diabetic animals with glibenclamide (0.25 mg/kg) or methanolic extract of *Bersama engleriana* significantly (p<0.05 - 0.01) improved the body weight during the entire period of treatment. This increase was more expressed in rats receiving the highest dose (600 mg/kg) of the methanolic extract (19.05%). On the contrary, aqueous extract of *Bersama engleriana* (300 and 600 mg/kg) did not show any improvement in the body weight (Table 2).

**Table 2:** Effects of *Bersama engleriana *on body weight, relative weights of liver and pancreas and, lipid profile on nicotinamide/streptozotocin-induced diabetic rats

**Groups**	**Body weight(g)**			**Relative organ weight (mg/g)**		**Lipid profile(mg/dl)**			
	**Initial**	**Final**	**% variation**	**Liver**	**Pancreas**	**TC**	**LDL-C**	**HDL-C**	**TG**
**Control**	210.06 ± 11.25	251.00 ± 10.57	19.48	33.86 ± 01.07	4.11 ± 0.50	98.06 ± 05.54	45.37 ± 03.93	36.15 ± 02.50	82.70 ± 05.81
**Untreated diabetic**	210.53 ± 12.36	203.00 ± 04.52	-3.57	11.23 ± 0.70^***^	1.37 ± 0.08^***^	153.53 ± 17.82^*^	118.8 3± 12.82^*^	12.44 ± 03.65^*^	96.39 ± 15.83
**Diabetic + Glibenclamide (0.25 mg/kg)**	217.88 ± 22.06	250.50 ± 10.57	14.97	13.46 ± 01.31^***^	0.96 ± 0.09^***^	134.77 ± 07.58^*^	88.09 ± 11.14^*#^	20.42 ± 02.84^*^	70.60 ± 07.87^#^
**Diabetic + Aqueous extract (300 mg/kg)**	250.85 ± 12.06	230.00 ± 06.35	-8.31	14.56 ± 0.69^***^	1.23 ± 0.15^***^	127.97 ± 02.07	89.27 ± 07.67^*#^	17.60 ± 02.05^*^	64.05 ± 09.61*^#^
**Diabetic + Aqueous extract (600 mg/kg)**	254.85 ± 15.50	237.33 ± 06.33	-6.87	15.70 ± 01.59^***^	1.60 ± 0.21^***^	130.62 ± 06.57^*^	76.12 ± 02.35^*##^	12.10 ± 01.51^*^	79.51 ± 11.64^#^
**Diabetic + Methanolic extract (300 mg/kg)**	199.41 ± 06.56	224.00 ± 11.69	12.33	15.69 ± 0.55^***^	2.42 ± 0.21^***##^	123.32 ± 12.46	89.48 ± 12.69^*#^	29.96 ± 07.28^#^	59.56 ± 11.39^***#^
**Diabetic + Methanolic extract (600 mg/kg)**	187.98 ± 09.24	223.67 ± 07.57	19.05	19.23 ±01.19^***###^	2.41 ± 0.16^***##^	100.75 ± 14.74^#^	59.19 ± 08.39^###^	32.97 ± 04.37^#^	56.01 ± 09.70^***##^

Values are means ± SEM; number of animals per group = 6.

*: p<0.05; *** p <0.001: significantly different compared to control.

^#^: p <0.05; ^##^: p <0.01; ^*###*^: p<0.001 significantly different compared to untreated diabetic.

The relative weights of pancreas and liver were also decreased in all diabetic animals when compared to controls. However with regard to diabetic animals, a dose-dependent increase in the relative weights of these vital organs was observed in the *Bersama engleriana* groups. It can be also noticed that the methanolic extract, especially the dose 600 mg/kg, produced more effects (p<0.001) than glibenclamide and aqueous extract (Table 2).

### Effects of Bersama engleriana on blood glucose

Table 3 shows the effects of aqueous and methanol extracts of the leaves of *Bersama engleriana* on blood glucose levels of Wistar rats after 4 weeks of continuous treatment. Sequential injections of nicotinamide and streptozotocin caused a significant increase (p <0.05 to 0.001) in blood glucose concentrations in all groups of rats compared with their respective baseline blood glucose (at the time of grouping) and to control values. At all-time points, blood glucose concentrations remained unchanged (p>0.05) in normal rats treated with distilled water. However, oral administration of the plant extracts as well as glibenclamide to diabetic rats provoked a week-dependent decrease (p<0.05 to 0.001) in blood glucose concentrations. Thus after 4 weeks of continuous treatment, the dose 600 mg/kg of the methanolic extract of *Bersama engleriana* produced the most alleviating effects (80.31%) compared to aqueous extract (600 mg/kg; 67.74%) and glibenclamide (58.65%). At equal dose, the methanolic extract was more efficient than the aqueous extract.

**Table 3:** Effects of *Bersama engleriana *on blood glucose in nicotinamide/streptozotocin-induced type 2 diabetic rats

**Groups**	**Fasting blood glucose level (mg/dl)**				
		**Week of treatment**			
	**At the time of grouping**	**Week 0**	**Week 1**	**Week 2**	**Week 4**
**Control**	97.80 ± 02.97	69.00 ± 07.40^a^	75.8 ± 09.05^a^	81.00 ± 04.25^a^	62.00 ± 03.45^a^
**Untreated diabetic**	97.40 ± 04.54	270.80 ± 81.14^a***^	244.40 ± 69.28^a***^	202.60 ± 55.54^a*^	203.00 ± 46.62^a**^
**Diabetic + Glibenclamide (0.25 mg/kg)**	86.40 ± 06.53	227.80 ± 83.62^a**^	146.40 ± 51.05^ad^	98.20 ± 09.28^bd^	94.20 ± 11.20^bd^
**Diabetic + Aqueous extract (300 mg/kg)**	95.40 ± 05.08	156.60 ± 20.76^#a^	121.80 ± 06.80^#a^	109.60 ± 07.15^a^	96.60 ± 02.67^a^
**Diabetic + Aqueous extract (600 mg/kg)**	91.00 ± 06.47	303.80 ± 41.86^a***^	183.60 ± 33.96^bd^	119.00 ± 04.63^cd^	98.00 ± 07.52^c^
**Diabetic + Methanolic extract (300 mg/kg)**	90.20 ± 04.18	181.80 ± 48.41^a*^	114.00 ± 15.71^#a^	86.60 ± 07.35^#a^	83.60 ± 08.18^#a^
**Diabetic + Methanolic extract (600 mg/kg)**	90.20 ± 03.38	454.00 ± 10.93^a*** ###^	288.40 ± 45.75^b***^	167.60 ± 42.28^c^	89.40 ± 06.23^#c^

Values are means ± SEM; number of animals per group = 6.

On the same line, values assigned with the same letter are not significantly different.

Within a column, *: p<0.05; **: p <0.01; *** p <0.001: significantly different compared to control.

^#^: p <0.05; ^###^: p <0.001: significantly different compared to untreated diabetic.

### Effects of Bersama engleriana on lipid profile

The diabetic condition in rats (untreated diabetics) raised TC (36.13%), LDL-C (61.82%) and TG (14.20%) concentrations and lowered HDL-C level (65.59%) (p<0.05) compared to control rats (non diabetic rats). Treatment of nicotinamide/streptozotocin-induced diabetic animals with *Bersama engleriana* extracts and especially the methanolic extract produced opposite effects evidenced by a decrease (p<0.05) in serum TC (percentage of decrease: 300 mg/kg, 19.68%; 600 mg/kg, 34.38%) and LDL-C (percentage of decrease: 300 mg/kg, 24.70%; 600 mg/kg, 50.19%) and an increase in the HDL-C (percentage of increase: 300 mg/kg, 58.48%; 600 mg/kg, 62.27%) concentration when compared to untreated diabetic. A tendency to a decrease in TG concentrations was also observed with regard to diabetic rats receiving distilled water. In general, glibenclamide produced similar effects.

As above observed, the methanolic extract appeared to be more efficient than the aqueous extract at equal dose and, more potent at high dose (600 mg/kg) than glibenclamide (Table 3).

## Discussion

Association of nicotinamide and streptozotocin is being increasingly used for inducing a diabetes mellitus (DM) similar to human’s type 2 DM [6-9]. In this sequential combination, nicotinamide protects pancreatic beta cells, delaying their disappearance, improves their regeneration and controls blood glucose parameters [13] whereas streptozotocin has toxic effect resulting in increased formation of free radicals that alter the plasma membrane of β cells and fragmented DNA [14]. The moderate hyperglycemic state (≥126 mg/dl) recorded in rats throughout the present experiment confirms the real diabetic status of the animals used in the present study.

In type 2 diabetic rats treated with *Bersama engleriana* extracts, a significant decrease (p<0.05) in blood glucose concentrations was observed when compared to respective baseline values (week 0). These results further support the hypoglycemic activity of *Bersama engleriana* previously reported in sugar overloaded normal rats [3]. It is generally believed that most of the medicinal plants with antidiabetic potentials have been found to contain a variety of substances responsible for the reported activities [15]. Thus, phytochemical tests revealed the presence of triterpenes, steroids, saponins and phenolic compounds in *Bersama engleriana* extracts. It has been demonstrated that triterpenes and phenols stimulate insulin secretion through their antioxidant activities [16,17]. Oxidative stress has been shown to play a key role in the causation of diabetes. Streptozotocin produces oxygen radicals in the body, which cause pancreatic injury and could be responsible for increased blood sugar as well as lipid peroxidation. As such, antioxidants may have a role in the alleviation of diabetes [18]. From the results obtained in this work, it could be proposed that *Bersama engleriana* may enhance the antioxidant defense against reactive oxygen species produced under hyperglycemic condition and this could protect beta-cells against loss, and exhibit antidiabetic activity. The *in vitro* antioxidant property of *Bersama engleriana* previously reported by [5] using extracts from the roots, stem bark, leaves and wood of this medicinal plant could be of great importance in the understanding of this suggested *in vivo* antioxidant activity of *Bersama engleriana*. It is a well-established fact that many medicinal plants possess antioxidant potentials which might be helpful under diabetic conditions [19-21]. Results from this work also indicate that the methanolic extract, especially the dose 600 mg/kg, produced more alleviating effects. This observation confirms the fact that methanol extracts of plants are generally known for their high contents in chemical compounds capable of producing biological activities [22].

With regard to the lowering blood glucose concentrations, it could be proposed that *Bersama engleriana* may act by (1) stimulating insulin secretion similarly to glibenclamide [23], (2) triggering progressive regeneration of the damaged β cells after sequential injection of nicotinamide and streptozotocin or (3) potentiating glucose uptake and use by various tissues [17,24,25]. The improvements observed in the body weight as well as in the relative weights of the pancreas and liver of diabetic animals after plant extract applications further support these proposed pancreatic and extra-pancreatic mechanisms of action of *Bersama engleriana*[26-29].

As one of the complications that followed diabetic hyperglycemia is dyslipidemia, the serum lipid profile of rats was evaluated in this study. As expected, untreated diabetic animals showed a significant increase in serum TC, LDL-C and TG concentrations against low levels of HDL-C [27,30,31]. This increase in serum lipids is mainly due to the increased fatty acid mobilization from adipose tissue. Since insulin has an inhibitory action on HMG-CoA reductase (3-hydroxy-3-methyl-glutaryl coenzyme A reductase), the key enzyme in cholesterol biosynthesis [32], insulin deficiency or insulin resistance may therefore be responsible for hyperlipidemia. Treatment of type 2 diabetic rats with *Bersama engleriana* extracts, especially its methanolic extract (600 mg/kg), reversed although not completely dyslipidemia as evidenced by the significant decrease (p<0.05) in TC, LDL-C and TG coupled to the increase in HDL-C (p <0.05). These alleviating effects clearly denote the antihyperlipidemic potential of *Bersama engleriana*[33], and may also account in the improvement of liver weight as above observed. It could also be suggested that this antihyperlipidemic effects of *Bersama engleriana* pass through a decrease in intestinal cholesterol absorption or a decrease in the biosynthesis of cholesterol specifically by decreasing the activity of HMG-CoA reductase inhibitors [34].

## Conclusions

From this study, we can conclusively state that, *Bersama engleriana* extract and especially the methanolic extract possesses antidiabetogenic properties and beneficial effects on diabetic hyperlipidemia. All these beneficial effects on this rat model could be due to the bioactive components revealed in the *Bersama engleriana* extracts such as triterpenes and phenols.

## Competing interest

We declare that we have no conflict of interest.

## Authors’ contributions

WP conceived the project and wrote the final draft of the protocol. WP and NTB did the literature search and assist in the methodology. WP, AJHG, MCU and WNM contributed to the laboratory work, data analysis and data interpretations. AK was responsible for the overall supervision. PW, AJHG and WNM wrote the paper with input from all the authors. All authors read and approved the final manuscript.

## Pre-publication history

The pre-publication history for this paper can be accessed here:

http://www.biomedcentral.com/1472-6882/12/264/prepub
